# Amyloid Beta and MicroRNAs in Alzheimer’s Disease

**DOI:** 10.3389/fnins.2019.00430

**Published:** 2019-05-03

**Authors:** Nnana Amakiri, Aaron Kubosumi, James Tran, P. Hemachandra Reddy

**Affiliations:** ^1^Department of Internal Medicine, Texas Tech University Health Sciences Center, Lubbock, TX, United States; ^2^Garrison Institute on Aging, Texas Tech University Health Sciences Center, Lubbock, TX, United States; ^3^Garrison Institute on Aging, South West Campus, Texas Tech University Health Sciences Center, Lubbock, TX, United States; ^4^Department of Cell Biology and Biochemistry, Texas Tech University Health Sciences Center, Lubbock, TX, United States; ^5^Department Pharmacology and Neuroscience, Texas Tech University Health Sciences Center, Lubbock, TX, United States; ^6^Department of Neurology, Texas Tech University Health Sciences Center, Lubbock, TX, United States; ^7^Department of Speech-Language and Hearing Clinics, Texas Tech University Health Sciences Center, Lubbock, TX, United States; ^8^Department of Public Health, Graduate School of Biomedical Sciences, Texas Tech University Health Sciences Center, Lubbock, TX, United States

**Keywords:** microRNA, Alzheimer’s disease, amyloid beta, mitochondria, aging

## Abstract

Alzheimer’s disease (AD) is a progressive mental illness characterized by memory loss and multiple cognitive impairments. In the last several decades, significant progress has been made in understanding basic biology, molecular mechanisms, and development of biomarkers and therapeutic drugs. Multiple cellular changes are implicated in the disease process including amyloid beta and phosphorylation of tau synaptic damage and mitochondrial dysfunction in AD. Among these, amyloid beta is considered a major player in the disease process. Recent advancements in molecular biology revealed that microRNAs (miRNAs) are considered potential biomarkers in AD with a focus on amyloid beta. In this article we discussed several aspects of AD including its prevalence, classifications, risk factors, and amyloid species and their accumulation in subcellular compartments. This article also discusses the discovery and biogenesis of miRNAs and their relevance to AD. Today’s research continues to add to the wealth of miRNA data that has been accumulated, however, there still lacks clear-cut understanding of the physiological relevance of miRNAs to AD. MiRNAs appear to regulate translation of gene products in AD and other human diseases. However, the mechanism of how many of these miRNAs regulate both the 5′ and 3′UTR of amyloid precursor protein (APP) processing is still being extrapolated. Hence, we still need more research on miRNAs and APP/amyloid beta formation in the progression and pathogenesis of AD.

## Introduction

Alzheimer’s disease (AD) is a progressive multifactorial neurodegenerative form of dementia. AD was first discovered in the late 19th and early 20th centuries by pioneer Alois Alzheimer who documented many patients sharing similar symptoms with similar deteriorations seen in the patients’ brain anatomy (Alzheimer et al., 1991). Due to the progressive degeneracy of the brain seen in this disease, AD is characterized by memory loss, numerous cognitive impairments, and changes in personality, thought, and behavior (Reddy et al., 2017). Early in the disease, AD has a substantial impact on one’s daily routine by affecting areas of the brain that control memory, executive cognition and visuospatial awareness. Personality, behavior, and language impairments tend to occur much later in the progression of AD. While AD greatly impacts patients, it also poses a significant emotional and financial burden on the patients’ family members.

Alzheimer’s disease is recognized as a disease especially prevalent in the elderly. Of almost 5 million people diagnosed with AD in the United States in 2010, approximately 4.1 million were age 75 or older (Hebert et al., 2013). Due to advances in healthcare the average lifespan of human beings has rapidly increased from 57.3 to 64.2 years for males and 58.2 to 68.5 years for females between 1990 and 2013 (Abubakar et al., 2015). Because the average lifespan of human beings is increasing, AD’s impact on our world today is increasing as well. Though an estimated 5.5 million people in the United States currently suffer from AD, this number is expected to increase to 13.8 million by 2050. Due to the growing aging population, AD has become a major public health concern, with global costs in 2018 estimated at around $1 trillion (World Alzheimer Report, 2018). Unfortunately, there is a lack of effective prevention methods and no cure to combat this growing health concern. Because of its growing worldwide medical and financial burden it’s important to understand the different ways AD presents itself, the disease at the molecular level, and the modifiable and non-modifiable factors contributing to the characteristic features of AD. Currently, there are no early detectable biomarkers and drugs that can delay and/or prevent the disease process.

The purpose of this article is to highlight recent developments in AD including, (1) amyloid beta (Aβ) toxicity, (2) abnormal APP processing, (3) discovery of miRNAs and their biogenesis, and (4) involvement of miRNAs in aging and AD, particularly with abnormal APP processing and Aβ formation. This article also summarizes miRNAs as potential biomarkers for AD.

## Types of Alzheimer’s Disease

Two forms of AD exist: sporadic AD and familial AD. Early-onset AD, known as familial AD, is an extremely uncommon form of the disease seen in one to two percent of all AD cases. It is defined as AD occurring before the age of 65. Late-onset AD, termed sporadic AD, is the overwhelmingly more common form of the disease affecting anyone at any age but usually occurring in those above the age of 65 (Dementia Australia, 2014). Familial AD is due to mutations in three major genes: amyloid beta precursor protein (APP) gene, presenilin1 (PSEN1) gene, and presenilin 2 (PSEN2) gene. Mutations in these genes induce the abnormal overproduction of amyloid-β, leading to familial AD. In sporadic AD, the cause is not as well understood. Sporadic AD is believed to be determined by a combination of genetic factors, environmental factors, and lifestyle (Piaceri et al., 2013).

## Risk Factors of AD

Several risk factors affect the probability of acquiring sporadic Alzheimer’s disease. This includes both modifiable and non-modifiable risk factors. Modifiable risk factors for sporadic AD involve type 2 diabetes or obesity, environmental factors such as stress, vascular diseases, depression, stroke, hypertension, traumatic brain injury, alcohol, smoking, exercise, and other lifestyle habits. Sporadic AD non-modifiable risk factors include genetic mutations, age, sex, or genetic polymorphisms. Apolipoprotein E4 (ApoE4) genotype is believed to be a non-modifiable risk factor present in both familial and sporadic AD. Genetic polymorphisms that can serve as risk factors in sporadic AD include but are not limited to alterations in sortilin-related receptor 1, clusterin, complement component receptor 1, CD2-associated protein (CD2AP), Ephrin type-A receptor 1 protein (EPHA1), and Membrane-spanning 4-domains subfamily A (MS4A6A/MS4A4E genes) (Herrera-Rivero, 2013). This article investigates the role miRNAs play in AD. Specifically, it delves into the interaction between miRNAs and Amyloid-β (Aβ) in order to show how miRNAs might induce early-onset familial AD and late-onset sporadic AD.

## Cellular Changes in Alzheimer’s Disease

Histopathological and morphological examination of postmortem AD brains and transgenic mouse models of AD revealed that multiple cellular changes are involved with the disease process including, loss of neurons, synaptic loss/damage, mitochondrial fragmentation, increased free radical production, mitochondrial DNA damage, proliferation of astrocytes and microglia, hormonal imbalance and altered neurotransmitter levels (e.g., decreased acetylcholine), in addition to neurofibrillary tangles and senile plaques (Selkoe, 2001; Reddy et al., 2010, 2012; Zhu et al., 2013; Swerdlow et al., 2014). These changes primarily were observed in learning and memory regions of the brain, including entorhinal cortex and spreads to the hippocampus, temporal cortex, frontoparietal cortex, and finally to subcortical nuclei (Reddy and McWeeney, 2006).

## Amyloid-β

The presence of extracellular amyloid plaques composed mainly of Aβ peptides in the brain is one of the hallmark features of AD. Identified in 1984 by scientists George Glenner and Caine Wong, Aβ are peptides of ∼40 amino acids long first described as a “novel cerebrovascular amyloid protein” (Glenner and Wong, 1984). Aβ is derived from a larger protein APP, one of the numerous mutated genes responsible for familial AD. In the non-AD pathway APP is predominantly cleaved by α-secretase, though the β-secretase pathway is part of normal physiology. In the disease-promoting pathway, the β-secretase pathway predominates. Cleavage by α- or β-secretase yields a protein fragment called secreted APP (sAPP) α or β, respectively. After β-secretase cleavage, sAPPβ is then cleaved by γ-secretase to yield Aβ. Cleavage by γ-secretase is inconsistent resulting in differences at the C-terminal end. These differences contribute to the existence of a wide array of Aβ species. The most numerous Aβ species include Aβ40 and Aβ42, or cleavage at the 40 and 42 positions, respectively (Murphy and LeVine, 2010).

## Amyloid Beta Species

Extensive past research has revealed that multiple Aβ species are present in the postmortem AD brains and in AD mouse models – these are Aβ1–36, 1–37, 1–38, 1–39, 1–40, and Aβ1–41, Aβ1–42, and Aβ1–43 (Kakuda et al., 2017). Among these Aβ40 is the most abundant form of Aβ species (Mori et al., 1992). While similar in structure, Aβ40 is composed of two protofilaments whereas Aβ42 is composed of only one protofilament. Both also comprise of the same number of Aβ molecules per cross-β repeat. Aβ42 is a slightly longer and less abundant form of Aβ species (Schmidt et al., 2009). Aβ42 is hydrophobic, fibrillogenic, and the main Aβ variant that accumulates in the brain of AD patients (Murphy and LeVine, 2010).

## Amyloid Beta – Pathogenic or Simply Bioproduct of Disease Process?

Though the exact pathogenic role of Aβ is unknown, the pathogenicity of Aβ is amplified when Aβ monomers become Aβ oligomers. Research shows that increasing Aβ plaque does not necessarily correlate with an amplified AD pathology. Aβ plaques can be present in both cognitively impaired individuals and those with normal cognitive function. Additionally, APP genetically modified mice have physiologic and behavioral abnormalities present before the appearance of Aβ plaque. This suggests that Aβ plaque buildup alone may not be responsible for the cognitive impairment associated with AD. Rather, smaller Aβ oligomers may play a more important role in the neurotoxicity seen in AD via its toxic effects on synapses in the brain (Mucke and Selkoe, 2012). Furthermore, it has also been shown that one can have very little plaque buildup but still demonstrate AD pathology (Erten-Lyons et al., 2009). This all is possible because the presence of small Aβ oligomers, not Aβ plaques, have been found to be primarily responsible for the impending cognitive impairment. Aβ oligomers are more likely to be pathogenic than Aβ plaques because they have a greater surface area to interact with the synapses of cells in the brain (Mucke and Selkoe, 2012). When analyzing mice with high Aβ plaque loads but deficient in oligomeric Aβ levels, researchers demonstrated that the mice did not experience any memory impairment (Lesné et al., 2008).

Evidence suggests that Aβ induces several different pathologies seen in AD. An example of said induction includes a role in stimulating tau protein tangles, the second hallmark feature of AD (Nussbaum et al., 2013). Another example of Aβ induced changes include the neuritic alterations and synaptic distortions seen greatest in cortical regions most proximal to Aβ plaques. The change from straight to coiled cortical dendrites is believed to decrease the efficiency of the transmission of signals along dendrites (Mucke and Selkoe, 2012).

## Amyloid Beta Accumulation in Subcellular Compartments and Cellular Toxicity

Several lines of evidence suggest that Aβ accumulates in multiple cellular compartments. Under normal conditions, Aβ was found in the small cytoplasmic granules in both neurites and perikarya. Minor portions of Aβ are colocalized with *trans*-Golgi network, Golgi-derived vesicles, early and late endosomes, lysosomes, synaptic vesicles, and mitochondria (Reddy and Beal, 2008; Zheng et al., 2012). It is well documented that toxicity of Aβ is dependent on the size, aggregation state, and diffusion of Aβ in subcellular compartments and at neuronal terminals (Sengupta et al., 2016).

## miRNAs and AD

Alzheimer’s disease research shows that miRNAs help regulate genes, protein expressions, and phenotypic changes in human diseases. Studies have shown miRNAs can contribute to the development of a variety of pathologic conditions. This includes a role in aging, cardiovascular disease, cancers, arthritis, cataracts, osteoporosis, diabetes/obesity, hypertension, and various neurodegenerative diseases such as AD, Huntington’s disease, Parkinson’s disease, amyotrophic lateral sclerosis, and schizophrenia (Kumar and Reddy, 2016; Reddy, 2017).

## Discovery of MicroRNAs

miRNAs are a large family of small RNAs, approximately 18–25 nucleotides in length, that play an integral role in the regulation of gene expression. Discovered in 1993, a non-protein-coding transcript named *lin-4* was identified as the first miRNA in the nematode *Caenorhabditis elegans*. *Lin-4* was shown to negatively regulate *lin-14* protein by complementary binding to its 3′UTR (Lee et al., 1993). In this species, the downregulation of *lin-14* by *lin-4* helps progress the embryo from the first to the second larval stage of development. Today thousands of miRNAs have been identified that anneal at the 3′ UTR of many human genes. Most recently, miRNAs have been shown to also have activity at the 5′ UTR of genes (Long et al., 2019). There are two classes of miRNAs: miRNAs associated with exons or the coding part of genes and those originating from introns, the non-coding part of genes. One-third of these miRNAs are found in the coding part of genes, and the remaining are in the non-coding regions (Abe and Bonini, 2013; Reddy, 2017).

## Biogenesis of MicroRNAs

Generation of miRNAs starts with the initial transcription by RNA polymerase II into a long primary transcript (pri-miRNA). In the nucleus, these pri-miRNAs are cleaved by two proteins Drosha and DiGeorge Syndrome Critical region 8 (DGCR8) proteins which dimerize together to form a functional microprocessor complex. These dimerized proteins cleave pri-miRNAs into precursor miRNAs (pre-miRNA) which are transported to the cytoplasm to be digested by proteins Dicer and TAR RNA binding protein (TRBP) to release a double-stranded miRNA duplex. A helicase unwinds the duplex assembly to form mature miRNA strands. One of these strands is usually degenerated while the other associates with the Ago2 protein to form miRNA-induced Silencing Complex (miRISC). Rarely, both can serve as mature functional miRNA. Functional miRNAs modulate gene activity by inhibiting miRNA cleavage or suppressing translation in the same way *lin-4* was previously described as suppressing *lin-14* translation in *C. Elegans* (Abe and Bonini, 2013).

**FIGURE 1:**
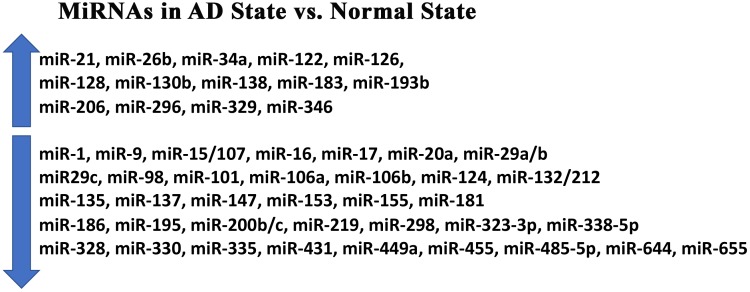
Major microRNAs in Alzheimer’s Disease. Up arrow indicates an increased levels of the miRNAs in Alzheimer’s disease. Down arrow indicates downregulated miRNAs in Alzheimer’s disease.

Most reported miRNAs are found in the human brain. Many of these miRNAs are responsible for synaptic functions, neurotransmitter release, synapse formation and neurite outgrowth. In diseased states such as AD, expression levels of several miRNAs are either downregulated or upregulated (Abe and Bonini, 2013; see Figure 1).

## Aging and MicroRNAs

Aging is the greatest risk factor for AD and many other neurodegenerative diseases. Within the aging process, cellular changes lead to increased disease vulnerability and mortality. Key to the aging process is cellular senescence, which miRNAs play a pivotal role in. Due to the critical role miRNAs play in cellular senescence and thus aging, they are pivotal in assessing one’s risk for AD. Some of the roles miRNAs largely influence include telomerase shortening, chronic inflammation, oxidative stress, mitochondrial dysfunction, DNA damage response, protein misfolding, stem cell impairment, and altered nutrient sensing (Reddy and Beal, 2008). Research is starting to show that miRNA’s have a wide array of functions that contribute to aging and cellular senescence (see Table 1).

**Table 1:** Summary of miRNAs related to aging and Alzheimer’s disease.

miRNA	Important role	Specific function	References
miR-1	Oxidative stress	Decreased in response to oxidative stress	Wu et al., 2015
miR-9	Tau regulation/amyloid-β regulation	Regulate MAPT splicing, altering 4R/3R ratio in neuronal cells. Downregulates serine palmitoyltransferase (SPT) which upregulates Aβ	Geekiyanage and Chan, 2011; Smith et al., 2011
miR-15b	Oxidative stress/amyloid-β regulation	Inhibits senescence-associated mitochondrial stress; downregulates BACE1 expression and hence Aβ by binding to the 3′ UTR of BACE1. Also inhibits NF-κB signaling, further inhibiting Aβ accumulation	Lang et al., 2016; Li and Wang, 2018
miR-16	Amyloid-β regulation	Directly targets APP by binding to its 3′ UTR, downregulating Aβ	Parsi et al., 2015
miR-17	Amyloid-β regulation	Directly targets APP by binding to its 3′ UTR, downregulating Aβ	Hébert et al., 2009
miR-20a	Oxidative stress/amyloid-β regulation	Directly targets APP by binding to its 3′ UTR, downregulating Aβ. SNP A454G can increase binding of miR-20a to APP mRNA, reducing APP’s expression. Upregulated in response to stress	Hébert et al., 2009; Zhang et al., 2014; Zhao et al., 2017
miR-21	Amyloid-β regulation	Inhibits Aβ-induced cell apoptosis by increasing PI3K, AKT, and GSK3β activity	Feng et al., 2018
miR-26b	Oxidative stress	Upregulated during oxidative stress	Absalon et al., 2013
miR-29a/b family	Amyloid-β regulation	Upregulation decreases BACE1 mRNA levels and hence Aβ by binding to the 3′ UTR of BACE1 and vice versa. Downregulates SPT which upregulates Aβ	Hébert et al., 2009; Geekiyanage and Chan, 2011
miR-29c	Amyloid-β regulation	Upregulation decreases BACE1 mRNA levels and hence Aβ by binding to the 3′ UTR of BACE1 and vice versa	Yang et al., 2015
miR-34a	Oxidative stress	Upregulated in liver of aging mice	Bai et al., 2011; Chen et al., 2011
miR-50	Oxidative stress	Altered in response to oxidative stress	Wu et al., 2015
miR-51	Oxidative stress	Altered in response to oxidative stress	Wu et al., 2015
miR-58	Oxidative stress	Altered in response to oxidative stress	Wu et al., 2015
miR-84	Oxidative stress	Altered in response to oxidative stress	Wu et al., 2015
miR-93	Oxidative stress	Targets glutathione-*S*-transferases, which normally protects from oxidative stress. Upregulated in liver of aging mice. Downregulated in liver of aging rats	Maes et al., 2008; Mimura et al., 2014
miR-98	Amyloid-β regulation/oxidative stress/mitochondrial dysfunction	Reduces the production of Aβ and increases oxidative stress and mitochondrial dysfunction via activation of the Notch signaling pathway by binding to HEY2	Chen et al., 2019
miR-101a-3p	Amyloid-β regulation	Directly targets APP by binding to its 3′ UTR, downregulating Aβ	Barbato et al., 2014; Lin et al., 2018
miR-106b	Amyloid-β regulation	Directly targets APP by binding to its 3′ UTR, downregulating Aβ	Hébert et al., 2009
miR-107	Amyloid-β regulation	Upregulation decreases BACE1 mRNA levels and hence Aβ by binding to the 3′ UTR of BACE1 and vice versa	Jiao et al., 2016
miR-122	Oxidative stress	Upregulated during oxidative stress	Song et al., 2015
miR-124	Amyloid-β regulation	Upregulation decreases BACE1 mRNA levels and hence Aβ by binding to the 3′ UTR of BACE1 and vice versa	An et al., 2017
miR-128	Oxidative stress/amyloid-β regulation	Downregulated during oxidative stress; downregulates PPAR-γ activating NF-κB which releases cytokines, further increasing Aβ production	Nidadavolu et al., 2013; Geng et al., 2018
miR-130b	Oxidative stress	Upregulated in response to oxidative stress	Zhang et al., 2014
miR-132	Tau regulation	Regulates MAPT splicing, altering 4R/3R ratio in neuronal cells; downregulates PTBP2	Smith et al., 2011
miR-135	Amyloid-β regulation	Directly targets APP by binding to its 3′ UTR, downregulating Aβ	Liu et al., 2014; Kumar and Reddy, 2016
miR-137	Tau regulation/amyloid-β regulation	Regulate MAPT splicing, altering 4R/3R ratio in neuronal cells. Downregulates SPT which upregulates Aβ	Geekiyanage and Chan, 2011; Smith et al., 2011
miR-138	Amyloid-β regulation	Promotes Aβ production by decreasing the expression of sirtuin 1 protein	Lu et al., 2019
miR-147	Amyloid-β regulation	Directly targets APP. SNP T171C can significantly inhibit the binding of miR-147 resulting in increased expression of APP and subsequent generation of Aβ	Delay et al., 2011
miR-153	Amyloid-β regulation	Directly targets APP by binding to its 3′ UTR, downregulating Aβ	Delay et al., 2011; Liang et al., 2012; Long et al., 2012
miR-155	Amyloid-β regulation	Directly targets APP by binding to its 3′ UTR, downregulating Aβ	Hébert et al., 2009
miR-181	Amyloid-β regulation	Downregulates SPT which upregulates Aβ, thus also downregulating Aβ	Geekiyanage and Chan, 2011
miR-183	Oxidative stress	Upregulated in response to oxidative stress	Li et al., 2009
miR-186	Amyloid-β regulation	Upregulation decreases BACE1 mRNA levels and hence Aβ by binding to the 3′ UTR of BACE1 and vice versa	Kim et al., 2016
miR-193b	Oxidative stress	Upregulated in response to oxidative stress	Zhang et al., 2014
miR-195	Amyloid-β regulation	Upregulation decreases BACE1 mRNA levels and hence Aβ by binding to the 3′ UTR of BACE1 and vice versa	Zhu et al., 2012
miR-200b	Amyloid-β regulation	Directly targets APP by binding to its 3′ UTR, downregulating Aβ; reduce Aβ secretion by promoting insulin signaling	Liu et al., 2014; Higaki et al., 2018
miR-200c	Oxidative stress/amyloid-β regulation	Upregulated in response to oxidative stress; Reduce Aβ secretion by promoting insulin signaling	Magenta et al., 2011; Higaki et al., 2018
miR-206	Aging	Downregulates expression of brain-neurotrophic factor (BDNF) which protects from cell death	Tian et al., 2014
miR-214	Oxidative stress	Targets glutathione-*S*-transferases, which normally protects from oxidative stress. Upregulated in liver of aging mice.	Maes et al., 2008
miR-219	Tau regulation	Directly binds to 3′UTR of tau mRNA, downregulating tau synthesis	Santa-Maria et al., 2015
miR-296	Oxidative stress	Upregulated in response to oxidative stress	Zhang et al., 2014
miR-298	Amyloid-β regulation	Upregulation decreases BACE1 mRNA levels and hence Aβ by binding to the 3′ UTR of BACE1 and vice versa	Boissonneault et al., 2009
miR-323-3p	Amyloid-β regulation	Directly targets APP by binding to its 3′ UTR, downregulating Aβ	Boissonneault et al., 2009; Delay et al., 2011; Liang et al., 2012
miR-328	Amyloid-β regulation	Upregulation decreases BACE1 mRNA levels and hence Aβ by binding to the 3′ UTR of BACE1 and vice versa	Boissonneault et al., 2009
miR-329	Oxidative stress	Upregulated in response to oxidative stress	Zhang et al., 2014
miR-330	Amyloid-β regulation/oxidative stress/mitochondrial dysfunction	Decreases expression of VAV1 via the MAPK pathway reducing Aβ production and alleviates oxidative stress and mitochondrial dysfunction	Zhou et al., 2018
miR-335	Oxidative stress/mitochondrial dysfunction	Downregulates antioxidant enzymes in the mitochondria	Bai et al., 2011
miR-338-5p	Amyloid-β regulation	Upregulation decreases BACE1 mRNA levels and hence Aβ by binding to the 3′ UTR of BACE1 and vice versa	Qian et al., 2019
miR-346	Amyloid-β regulation	Directly targets APP by binding to its 5′ UTR, downregulating Aβ	Long et al., 2019
miR-355	Oxidative stress	Altered in response to oxidative stress	Wu et al., 2015
miR-431	Amyloid-β regulation	When present, prevents Aβ-induced synaptic loss in cortico-hippocampal cells and prevents neurite degeneration	Ross et al., 2018
miR-449a	Oxidative stress	Downregulated in response to oxidative stress	Nidadavolu et al., 2013
miR-455	Oxidative stress	Downregulated in response to oxidative stress	Nidadavolu et al., 2013
miR-485-5p	Amyloid-β regulation	Upregulation decreases BACE1 mRNA levels and hence Aβ by binding to BACE1 exon 6	Faghihi et al., 2010
miR-644	Amyloid-β regulation	Directly targets APP by binding to its 3′ UTR, downregulating Aβ	Delay et al., 2011
miR-655	Amyloid-β regulation	Directly targets APP by binding to its 3′ UTR, downregulating Aβ	Delay et al., 2011
miR-669c	Oxidative stress/mitochondrial dysfunction	Targets glutathione-*S*-transferases, which normally protects from oxidative stress. Upregulated in liver of aging mice	Maes et al., 2008
miR-709	Oxidative stress/mitochondrial dysfunction	Targets glutathione-*S*-transferases, which normally protects from oxidative stress. Upregulated in liver of aging mice	Maes et al., 2008
miR-796	Oxidative stress	Altered in response to oxidative stress	Wu et al., 2015


Additional support for miRNA as a causal factor includes the observation of altered miRNA levels in those suffering or at risk for AD. The role of miRNAs in aging and AD is further summarized in Reddy et al. (2017). MiRNAs are directly involved with aging and cellular senescence by disrupting protein regulation. In addition, as shown in Table 1, a large number of miRNAs are associated with aging and cellular senescence. Further research is needed to better understand the relationship between age-related miRNAs in inducing AD and other neurodegenerative diseases.

## MicroRNAs and Abnormal APP Processing and Aβ Formation

Recent research has reinforced previously hypothesized mechanisms in which miRNAs induce Aβ production while highlighting more miRNAs that may work via these methods and perhaps revealing new mechanisms through which miRNAs operate. Table 2 shows altered miRNAs directly related to Aβ in AD while Figure 1 illustrates the ways in which these miRNAs are altered in the AD state compared to a normal physiologic state. Though their involvement is not well defined, miRNAs are thought to be a causal factor in Aβ formation.

**Table 2:** Summary of miRNAs regulating amyloid-β.

miRNA	Specific function	References
miR-9	Downregulates serine palmitoyltransferase (SPT) which upregulates Aβ, thus also downregulating Aβ	Geekiyanage and Chan, 2011
miR-15b	Inhibits senescence-associated mitochondrial stress; downregulates BACE1 expression and hence Aβ by binding to the 3′ UTR of BACE1. Also inhibits NF-κB signaling, further inhibiting Aβ accumulation	Li and Wang, 2018
miR-16	Directly targets APP by binding to its 3′ UTR, downregulating Aβ	Parsi et al., 2015
miR-17	Directly targets APP by binding to its 3′ UTR, downregulating Aβ	Hébert et al., 2009
miR-20a	Directly targets APP by binding to its 3′ UTR, downregulating Aβ	Hébert et al., 2009
miR-21	Inhibits Aβ-induced cell apoptosis by increasing PI3K, AKT, and GSK3β activity	Feng et al., 2018
miR-29a/b family	Upregulation decreases BACE1 mRNA levels and hence Aβ by binding to the 3′ UTR of BACE1 and vice versa. Downregulates SPT which upregulates Aβ	Hébert et al., 2009; Geekiyanage and Chan, 2011
miR-29c	Upregulation decreases BACE1 mRNA levels and hence Aβ by binding to the 3′ UTR of BACE1 and vice versa	Yang et al., 2015
miR-98	Reduces the production of Aβ via activation of the Notch signaling pathway by binding to HEY2	Chen et al., 2019
miR-101a-3p	Directly targets APP by binding to its 3′ UTR, downregulating Aβ	Barbato et al., 2014; Lin et al., 2018
miR-106b	Directly targets APP by binding to its 3′ UTR, downregulating Aβ	Hébert et al., 2009
miR-107	Upregulation decreases BACE1 mRNA levels and hence Aβ by binding to the 3′ UTR of BACE1 and vice versa	Jiao et al., 2016
miR-124	Upregulation decreases BACE1 mRNA levels and hence Aβ by binding to the 3′ UTR of BACE1 and vice versa	An et al., 2017
miR-128	Downregulated during oxidative stress; downregulates PPAR-γ activating NF-κB which releases cytokines, further increasing Aβ production	Geng et al., 2018
miR-135	Directly targets APP by binding to its 3′ UTR, downregulating Aβ	Liu et al., 2014; Kumar and Reddy, 2016
miR-137	Downregulates SPT which upregulates Aβ, thus also downregulating Aβ	Geekiyanage and Chan, 2011
miR-138	Promotes Aβ production by decreasing the expression of sirtulin-1 protein	Lu et al., 2019
miR-147	Directly targets APP. SNP T171C can significantly inhibit the binding of miR-147 resulting in increased expression of APP and subsequent generation of Aβ	Delay et al., 2011
miR-153	Directly targets APP by binding to its 3′ UTR, downregulating Aβ	Delay et al., 2011; Liang et al., 2012; Long et al., 2012
miR-155	Directly targets APP by binding to its 3′ UTR, downregulating Aβ	Hébert et al., 2009
miR-181	Downregulates SPT which upregulates Aβ, thus also downregulating Aβ	Geekiyanage and Chan, 2011
miR-186	Upregulation decreases BACE1 mRNA levels and hence Aβ by binding to the 3′ UTR of BACE1 and vice versa	Kim et al., 2016
miR-195	Upregulation decreases BACE1 mRNA levels and hence Aβ by binding to the 3′ UTR of BACE1 and vice versa	Zhu et al., 2012
miR-200b/c	Directly targets APP by binding to its 3′, downregulating Aβ; reduce Aβ secretion by promoting insulin signaling	Liu et al., 2014; Higaki et al., 2018
miR-298	Upregulation decreases BACE1 mRNA levels and hence Aβ by binding to the 3′ UTR of BACE1 and vice versa	Boissonneault et al., 2009
miR-323-3p	Directly targets APP by binding to its 3′ UTR, downregulating Aβ	Boissonneault et al., 2009; Delay et al., 2011; Liang et al., 2012
miR-328	Upregulation decreases BACE1 mRNA levels and hence Aβ by binding to the 3′ UTR of BACE1 and vice versa	Boissonneault et al., 2009
miR-330	Decreases expression of VAV1 via the MAPK pathway reducing Aβ production and alleviates oxidative stress and mitochondrial dysfunction	Zhou et al., 2018
miR-338-5p	Upregulation decreases BACE1 mRNA levels and hence Aβ by binding to the 3′ UTR of BACE1 and vice versa	Qian et al., 2019
miR-346	Directly targets APP by binding to its 5′ UTR, downregulating Aβ	Long et al., 2019
miR-431	When present, prevents Aβ-induced synaptic loss in corticohippocampal cells and prevents neurite degeneration by silencing Kremen1	Ross et al., 2018
miR-485-5p	Upregulation decreases BACE1 mRNA levels and hence Aβ by binding to BACE1 exon 6	Faghihi et al., 2010
miR-644	Directly targets APP by binding to its 3′ UTR, downregulating Aβ	Delay et al., 2011
miR-655	Directly targets APP by binding to its 3′ UTR, downregulating Aβ	Delay et al., 2011


Many miRNAs have been documented as directly regulating APP expression levels on its 3′ UTR. Recently, Lahiri’s group reported a novel activity of miR-346: specifically, that it targets the APP mRNA 5′-UTR to upregulate APP translation and Aβ production (Long et al., 2019). This upregulation is reduced but not eliminated by the knockdown of the argonaute 2 protein. The target site for miR-346 overlaps with active sites for an iron-responsive element (IRE) and an interleukin-1 (IL-1) acute box element. IREs interact with iron response protein1 (IRP1), an iron-dependent translational repressor. In primary human brain cultures, miR-346 activity required chelation of Fe. In addition, miR-346 levels are altered in late-Braak stage AD. Thus, miR-346 plays a role in upregulation of APP and increased formation of Aβ in AD brains (Long et al., 2019).

In addition, 3′ UTR of APP gene is also largely involved with APP regulation. Recently, a number of single nucleotide polymorphisms located in the 3′UTR of *APP* have been found in AD patients with family history of dementia. Delay et al. (2011) extensively studied the role of polymorphisms in 3′ UTR in regulating APP expression regulation and miRNA function. Their bioinformatics analysis identified 12 putative miRNA bindings sites located in or near the *APP* 3′UTR variants T117C, A454G, and A833C. Among those candidates, seven miRNAs, including miR-20a, miR-17, miR-147, miR-655, miR-323-3p, miR-644, and miR-153 could regulate APP expression *in vitro* and under physiological conditions in cells. Using luciferase assay, they demonstrated that the T117C variant inhibited miR-147 binding, whereas the A454G variant increased miR-20a binding, consequently having opposite effects on APP expression. Further, research is still needed to understand the roles of the APP gene’s 3′ UTR in relation to miRNAs.

Lu’s lab highlighted a mechanism used by miR-138 involving the miRNA, sirtulin-1 protein, and circular RNA. They describe how miR-138 downregulates the Sirtulin-1 protein which induces Aβ production. This can then be reversed by inhibition of miR-138 via circular RNA, specifically circular HDAC9 (Lu et al., 2019).

Past research has shown β-site amyloid precursor protein cleaving enzyme, (BACE)-1, to be required for the cleavage of amyloid precursor protein (APP) to generate Aβ. This cleavage stimulates the nuclear transcription factor (NF-κB) leading to the secretion of inflammatory cytokines (Li and Wang, 2018). Many miRNAs, as seen in Table 2, inhibit BACE1 mRNA levels so in the AD state they are downregulated, contributing to elevated Aβ production. Geng’s lab illuminated how some miRNAs can activate PPAR-gamma which also leads to the activation of NF-κB further leading to the release of cytokines and Aβ production (Geng et al., 2018).

Several miRNAs also act to increase Aβ production via the Notch signaling pathway. Specifically, miRNAs are shown to inhibit HEY2 protein levels which inactivates the Notch signaling pathway. This Notch pathway is responsible for suppressing Aβ production among many other functions in the non-AD state (Chen et al., 2019).

Another of the many mechanisms by which miRNAs are proposed to regulate amyloid-beta production is through insulin signaling (Higaki et al., 2018). Higaki’s research detailed how the miR-200 family inhibit the expression of ribosomal protein S6 kinase B1 (S6K1), a downstream effector of mammalian target of rapamycin (mTOR). The protein m-TOR normally would suppress insulin release which leads to insulin resistance, typically seen in AD brains.

In Feng’s lab, they highlighted another mechanism through which miRNAs can alter amyloid-β levels. They show how miR-21 can inhibit Aβ-induced apoptosis (Feng et al., 2018). Normally, Aβ would be able to increase Bax and inhibit Bcl-2 protein levels. Both actions would compromise the cell membrane of cells and lead to eventual cell death. MiR-21 is upregulated in AD to invoke its protective effect by increasing the activity of the phosphatidylinositol 3-kinase/Protein Kinase B (also known as PI3K/AKT) pathway and increase glycogen synthase kinase-3β (GSK-3β) levels. These signaling pathways diminish Aβ-induced cell death.

MiRNA dysregulation is present in both tau and Aβ pathologies. Though both pathologies can lead to the AD state, it’s not clear which changes miRNA levels and what results from these changes. Interestingly, four miRNAs (miR142a-5p, miR146a-5p, miR155-5p, and miR-455-5p) have been shown to be commonly upregulated in transgenic tau and APP mice in the AD state (Sierksma et al., 2018). Through Sierksma’s investigation, it is shown that these miRNAs are not responsible for inducing cognitive disturbances because when they are upregulated in healthy mice the miRNAs do not induce AD-related cognitive disturbances. Henceforth, these miRNAs may be upregulated as part of a protective mechanism in the AD brain. Previous literature has shown several of these miRNAs to be downregulated in the AD brain, further supporting this hypothesized protecting role in the AD brain (see Table 1).

## MicroRNAs as Potential Biomarkers

A potential biomarker for early diagnosis of AD are miRNAs found in circulatory biofluids. Circulatory biofluids include cerebrospinal fluid (CSF), extracellular fluid (ECF), peritoneal fluid, pleural fluid, seminal fluid, bronchial secretions, breast milk, serum, plasma, and various other bodily fluids. The stability and abundance of circulatory miRNAs contribute to their ability to be a potential diagnostic biomarker for disease. The levels of miRNAs in these fluids change with changes in body physiology naturally seen in aging, changing diets, and pathologic states. It is believed that in addition to brain imaging, miRNA analysis of a blood sample might serve as a promising tool when attempting to diagnose whether a person has or is likely to contract cognitive impairment due to AD (Kumar and Reddy, 2016).

Various tests have revealed that disrupting miRNA biogenesis causes neurodegeneration. For example, a neurodegenerative disease is likely to result if one disrupts the Dicer protein which cleaves pre-miRNA into a double-stranded miRNA duplex. Studies like this show that miRNAs strongly impact long-term brain integrity (Abe and Bonini, 2013). Though a disruption in miRNA biogenesis is thought to be linked to the onset of many neurodegenerative diseases, outside of AD, very few studies have shown miRNAs use as a potential biomarker (Reddy, 2017).

While still much can be ascertained on the identity of miRNAs as peripheral biomarkers for Alzheimer’s disease in general, their presence and identity might help clarify what stage of AD the patient is in. Age is the biggest risk factor for AD so recently Ryan et al. (2018) tracked how the expression of miRNAs changed over time. Eight miRNAs were significantly affected by age either prior to or during Aβ plaque deposition. There experiments helped reinforce the notion that biological processes that lead to AD change over time and highlights potential pre-symptomatic biomarkers that are found to be distinct from the miRNAs highlighted in Table 2 which generally highlight the dysregulation of miRNA in the symptomatic AD state.

## Conclusion and Future Directions

Alzheimer’s disease is a progressive neurodegenerative disease characterized by memory loss and multiple cognitive impairments. With growing aging population, the prevalence of AD is high and is a major health concern in the society. Although tremendous progress has been made in understanding basic biology, molecular mechanisms of disease and therapeutics, we still do not have non-invasive early detectable biomarkers and drugs that can delay and/or prevent disease process. Several years of intense research revealed that multiple cellular changes are implicated in disease progression and pathogenesis, including synaptic damage, mitochondrial dysfunction, increased proliferation of astrocytes and microglia, in addition to the presence of core amyloid beta and phosphorylation of tau in AD affected regions of the brain. Amyloid beta is considered a major player in the disease process because of its relevance to both early-onset familial and late-onset sporadic AD. Recent research also revealed that microRNAs are considered as potential, non-invasive peripheral biomarkers in aging and other age-related neurodegenerative diseases such as AD. In this article, we discussed several aspects, including altered levels of miRNAs with aging, AD, particularly related to amyloid beta in disease process. In recent years, a wealth of miRNA data has been accumulated, but there is no single microRNA that regulate APP processing and amyloid beta formation and cognitive decline. It is possible that several miRNAs are involved in APP processing – some regulate 5′ UTR and others 3′ UTR of APP gene. To understand the biology of miRNAs and how they regulate both 5′- and 3′-UTR of APP processing, we still need more research on miRNAs and APP/amyloid beta formation in the progression and pathogenesis of AD.

## Author Contributions

PR contributed in designing the project. NA, PR, JT, and AK contributed in writing and checking and finalizing the manuscript.

## Conflict of Interest Statement

The authors declare that the research was conducted in the absence of any commercial or financial relationships that could be construed as a potential conflict of interest.
